# An Alteration of Lymphocytes Subpopulations and Immunoglobulins Levels in Patients with Diabetic Foot Ulcers Infected Particularly by Resistant Pathogens

**DOI:** 10.1155/2016/2356870

**Published:** 2016-12-05

**Authors:** Vladimíra Fejfarová, Alexandra Jirkovská, Michal Dubský, Frances Game, Jana Vydláková, Alena Sekerková, Jana Franeková, Monika Kučerová, Ilja Stříž, Vladimír Petkov, Robert Bém, Veronika Wosková, Andrea Němcová, Jelena Skibová

**Affiliations:** ^1^Diabetes Centre, Institute for Clinical and Experimental Medicine, Prague, Czech Republic; ^2^Diabetes Unit, Derby Hospitals NHS Foundation Trust, Derby, UK; ^3^Department of Clinical and Transplant Immunology, Institute for Clinical and Experimental Medicine, Prague, Czech Republic; ^4^Department of Clinical Biochemistry, Institute for Clinical and Experimental Medicine, Prague, Czech Republic; ^5^Department of Clinical Microbiology, Institute for Clinical and Experimental Medicine, Prague, Czech Republic

## Abstract

The* aim* of our study was to analyse immune abnormalities in patients with chronic infected diabetic foot ulcers (DFUs) especially those infected by resistant microorganisms.* Methods*. 68 patients treated in our foot clinic for infected chronic DFUs with 34 matched diabetic controls were studied. Patients with infected DFUs were subdivided into two subgroups according to the antibiotic sensitivity of causal pathogen: subgroup S infected by sensitive (*n* = 50) and subgroup R by resistant pathogens (*n* = 18). Selected immunological markers were compared between the study groups and subgroups.* Results*. Patients with infected chronic DFUs had, in comparison with diabetic controls, significantly reduced percentages (*p* < 0.01) and total numbers of lymphocytes (*p* < 0.001) involving B lymphocytes (*p* < 0.01), CD4+ (*p* < 0.01), and CD8+ T cells (*p* < 0.01) and their naive and memory effector cells. Higher levels of IgG (*p* < 0.05) including IgG1 (*p* < 0.001) and IgG3 (*p* < 0.05) were found in patients with DFUs compared to diabetic controls. Serum levels of immunoglobulin subclasses IgG2 and IgG3 correlated negatively with metabolic control (*p* < 0.05). A trend towards an increased frequency of IgG2 deficiency was found in patients with DFUs compared to diabetic controls (22% versus 15%; NS). Subgroup R revealed lower levels of immunoglobulins, especially of IgG4 (*p* < 0.01) in contrast to patients infected by sensitive bacteria. The innate immunity did not differ significantly between the study groups.* Conclusion*. Our study showed changes mainly in the adaptive immune system represented by low levels of lymphocyte subpopulations and their memory effector cells, and also changes in humoral immunity in patients with DFUs, even those infected by resistant pathogens, in comparison with diabetic controls.

## 1. Introduction

During the process of infection, after the engagement of innate immunity into the defence system, adaptive immunity is activated. This type of immunity consists of dual branches of cellular and humoral immunity. The principal effectors of cellular immunity are T lymphocytes, while the principle effectors of humoral immunity are B lymphocytes [1]. The activation of adaptive immunity is mediated by the production of antibodies by B lymphocytes that are able, for example, to block the adhesion of pathogens on mucosal surfaces, agglutinate bacteria, or to neutralise pathogens in blood circulation and subsequently activate complement [2]. The antigens are then eliminated by T cells or in concert with specific antibodies further produced by B cells [1].

Abnormal cell-mediated immunity, including changes in main immunoglobulins as well as their subclasses that play a specific role in the immune response cascades, could further reduce immune functions [3, 4]. Namely, IgG subclasses IgG1 and IgG3 are complement activators via their binding to the protein antigens of the pathogens. IgG2 usually mediates immune response to polysaccharide (bacterial) antigen and opsonisation of encapsulated bacteria [3]. Subclass IgG4 binds to protein antigens of Gram-positive bacteria and activates phagocytic cells. IgG subclass deficiencies lead to the alteration of immune defence against microbial pathogens and thus increase the risk of repeated infections [5, 6]. Alterations of IgG subclasses have been described specifically in immunocompromised patients [7].

The immune system has been studied in patients with diabetes mellitus but with conflicting results [8–11]. In the face of chronic hyperglycaemia (predominantly of more than 10 mmol/L), alterations of several steps of phagocytosis [12–14] including oxidative burst [15, 16] and occasionally abnormalities of selected lymphocyte subpopulations [17] as well as immunoglobulins [18] have been described in both in vivo and in vitro studies.

Therefore, we could hypothesised that the impairment of immune system function could be present also in patients with diabetic foot ulcers (DFUs), in whom infection related complications occur quite frequently and could lead to lower limb amputation [19, 20]. In our previous study, we investigated possible changes of the immune system in patients with chronic DFUs. The results of this study revealed mild immunological changes characterised by the activation of the inflammatory response such as increased leucocyte and neutrophil counts, CRP, and IgA levels. However, more serious immunodeficiencies of adaptive or innate immunity have not been observed in such risk group of diabetic patients to date [21, 22].

The aim of our current study was to analyse in detail the presence of possible immunological abnormalities in humoral as well as in cell-mediated immunity in patients with more severe, chronically infected DFUs and the potential relationship between immunological alterations and glycaemic control. In addition, we analysed differences in selected immune parameters between the patients with ulcers infected by sensitive pathogens and those infected by resistant bacterial strains recruited presumably from repeated chronic infections and antibiotic therapy [23, 24] in whom we hypothesised an immunocompromisation.

## 2. Subjects, Materials, and Methods

### 2.1. Subjects

68 patients with type 1 and type 2 diabetes mellitus aged between 30 and 70 years with infected DFUs were consecutively included into our cross-sectional study. Control group was formed by 34 patients matched by age, gender, and type of diabetes but without a history of diabetic foot disease.

We included those patients with DFUs who had been treated according to current guidance by the IWGDF [25] in our out-patient foot clinic for their DFUs for at least six weeks, were classified as Texas II-III/B-D, and had mild or moderate infection as defined by the PEDIS system [26]. The presence of infection was confirmed by clinical findings and/or elevated laboratory markers of inflammation [25]; osteomyelitis was defined clinically, by X-ray, positive microbial findings, and/or laboratory signs of infection [25].

Before inclusion, patients with DFUs had to have at least two positive wound cultures from swabs taken from the deep tissue after local debridement [27]. Swabs were cultured by standard microbial methods. Resistance to antibiotics was tested by sets of antibiotics composed on the basis of previous analysis of resistance at the Institute for Clinical and Experimental Medicine and upon the recommendations of the National Reference Laboratory on Antibiotics, National Institute of Health, Prague, Czech Republic. Microbial resistance to different types of antibiotics was determined by disc diffusion test [28, 29]. Minimum inhibitory concentration (MIC) was evaluated only in special cases by broth microdilution testing on microplates or by using the gradient method (*E*-test) [30]. Resistant pathogens were defined as those resistant to all oral antibiotics or produced enzymes causing microbial resistance such as ESBL (broad-spectrum beta-lactamase), AMPC (beta-lactamase C), and Mec gen. Sensitive microorganisms were considered those with an acceptable susceptibility to tested antibiotics. Our Department of Clinical Microbiology works with EUCAST methodology to interpret the results of sensitivity using clinical breakpoints according to the current version. To identify resistance phenotypes (determination of resistant/sensitive pathogens) we also used methods developed by the National Reference Laboratory on Antibiotics, National Institute of Health, Prague, Czech Republic. Patients with critical limb ischemia (Doppler Ankle/Brachial Index <0.6 and/or Doppler Toe Pressure <50 mmHg and/or Transcutaneous Oxygen Pressure (TcPO_2_) <30 mmHg and/or advanced arterial stenosis or occlusion requiring endovascular/surgical revascularisation based on invasive arterial assessment, angiography, CT-, or MR-angiography), with no option critical limb ischemia, advanced renal insufficiency (based on chronic kidney disease classification of 4th and 5th stage), active cancer, signs of acute infection, treatment with corticosteroids or other immunosuppressive drugs, post-organ transplant, with known immune dysfunction, hepatic failure, or malnutrition were excluded.

All patients with infected chronic DFUs included into our study were further divided into two subgroups: patients from whom sensitive microbial strains were isolated (subgroup S, *n* = 50) and those from whom resistant microbes (subgroup R, *n* = 18) were isolated on at least one occasion. These subgroups did not differ significantly in age, TcPO_2_, type and duration of DFUs, the incidence of osteomyelitis, or DFUs characteristics (depth, area).

The local ethics committee approved our study. Prior to enrolment into the study, each patient signed an informed consent form.

### 2.2. Methods

#### 2.2.1. Biochemical Analyses

The following were measured in all participants: blood glucose level (by spectrophotometry; Abbott Architect, USA), creatinine (detected enzymatically; Abbott Architect, USA), and glycosylated haemoglobin (HbA1c: normal values 20–42 mmol/mol; by HPLC Method; Tosoh G8, Japan).

#### 2.2.2. Inflammatory Markers

From laboratory markers of infection were assessed CRP (determined turbidimetrically; Abbott Architect, USA), procalcitonin (by electrochemiluminescence; ECLIA, Cobac 6000, Roche, Switzerland), and blood cell counts (by spectrophotometry; SYSMEX, Japan).

#### 2.2.3. Measures of Innate Immunity

Complements represented by C3, C4 (by immunonephelometry; Abbott Architect, USA), the absolute amounts and percentages of NK cells (CD16/56+ cells), and CD14+HLA-DR cells (monocytes, which serve as an important prognostic factor for the progression of an infection, especially in septic stage) were measured. NK cells and CD14+HLA-DR cells were determined together with subpopulations of lymphocytes (see below) by flow cytometry.

Phagocytosis was defined by the percentage of phagocytic cells and FAGSI (phagocyte stimulation index). Phagocytosis was assessed by the FagoFlowEx® Kit (Exbio Prague, Czech Republic). Phagocytic activity of granulocytes was tested by measuring the respiratory (oxidative) burst after their stimulation with* E. coli* bacteria in human heparinized whole blood using flow cytometry.

#### 2.2.4. Measures of Adaptive Immunity

CD3+, CD4+, CD8+, naive inactive (CD4+CD45RA+CD62L+), memory inactive (CD4+CD45RA−CD62L+), naive effector (CD4+CD45RA+CD62L−) and memory effector CD4+ T lymphocytes (CD4+CD45RA−CD62L−) and naive inactive (CD8+CD45RA+CD62L+), memory inactive (CD8+CD45RA−CD62L+), naive effector (CD8+CD45RA+CD62L−), and memory effector CD8+ T lymphocytes (CD8+CD45RA−CD62L−) were assessed by flow cytometry. Also we assessed their indexes counted as naive/memory cells.

During flow cytometry venous blood samples were collected into sterile tubes containing EDTA. Lymphocytes from peripheral blood (100 *µ*L; approximately 1 × 10^6^ cells) were labelled with a 4-color monoclonal antibody panel CYTO-STAT tetraChrome CD45-FITC (clone: B3821F4A)/CD56-RD1 (clone: N901/NKH1)/CD19-ECD (clone: J3-119)/CD3-PC5 (clone: UCHT1) + CD16-PE (clone: 3G8) and CD45-FITC (clone: B3821F4A)/CD4-RD1 (clone: SFCI12T4D11)/CD8-ECD (clone: SFCI21Thy2D3)/CD3 (clone: UCHT1) (all Beckman Coulter, Brea, CA). The specific antibody panels used for T effector cells consisted of anti-CD4-PE (clone: 13B8.2), anti-CD8-PC7 (clone: SFCI21Thy2D3), anti-CD62L-PC5 (clone: DREG56), and anti-CD45RA-FITC (clone: ALB11) (all Beckman Coulter, Brea, CA). The specific antibody panels used for activation of CD14+ cells consisted of anti-CD14 (clone: RM052) and anti-HLA-DR-PE (clone: Immu357) (Beckman Coulter, Brea, CA). Following staining, samples were analysed using an FC 500 flow cytometer (Beckman Coulter) with CxP and Kaluza software (Beckman Coulter). Flow cytometric analyses were performed with at least 100 gated events.

Serum levels of immunoglobulins (IgM, IgA, and IgG, diagnosed by immunoturbidimetry; Abbott Architect, USA) and IgG subclasses (determined by immunonephelometry; Immage/Immage 800, Beckman Coulter, USA) were also measured. In addition to this, we also compared the number of patients with deficits, particularly in IgG subclasses. Deficiencies were defined as serum levels of certain immunoglo-bulin or its subclass below physiological ranges that were determined by several studies [31] and the Producer Beckman Coulter.

### 2.3. Statistics

The characteristics of patients, laboratory markers of infection, and selected measures of innate and adaptive immunity, including lymphocyte subpopulations and IgG subclasses, were compared between the study groups and subgroups. Data analyses were performed using BMDP software (PC 90). Descriptive data were presented as means ± SD; differences between all study groups were determined using *t*-test, Mann–Whitney test, and multiple comparisons for Kruskal-Wallis test. Differences were considered as statistical significant with *p* < 0.05. The Spearman rank correlation coefficient was used to determine any significant correlation between assessed data.

## 3. Results

Patients with infected DFUs did not differ significantly in basic characteristics from the diabetic controls apart from higher serum creatinine levels (Table 1). Study patients with DFUs had median of DFU duration 7.5 months (range 1.5–48 months) and median of ulcer area 1 cm^2^ (range 0.04–43.4 cm^2^). 66.2% of patients had DFUs of Texas classification IIB/D and 33.8% of Texas classification IIIB/D. Chronic osteomyelitis was present in 41.2% of patients with DFUs.

Total numbers of leukocytes (7.7 ± 1.9 versus 8.5 ± 2.4 × 10^9^/L; NS), neutrophils (5.1 ± 1.5 versus 5.3 ± 1.8 × 10^9^/L; NS), and other laboratory markers of infection instead of CRP (Table 1) did not differ significantly between patients with DFUs and diabetic controls. There were no changes in measures of innate immunity except for lower absolute numbers of NK cells between the two study groups (Table 2).

Differences were observed predominantly in measures as of cellular as of humoral branch arm of adaptive immunity. Reductions of percentages and absolute values of total lymphocytes and decreased absolute numbers of almost all types of lymphocytes subpopulations including B lymphocytes, CD4+, CD8+ T lymphocytes, and their effector and memory cells (Table 3) as well as changes in humoral immunity were found in patients with infected DFUs (Table 4). From the evaluated immunological measures only IgG2 (*r* = −0.2008; *p* < 0.05) and IgG3 (*r* = −0.1972; *p* < 0.05) significantly negatively correlated with HbA1c.

**Figure 1:**
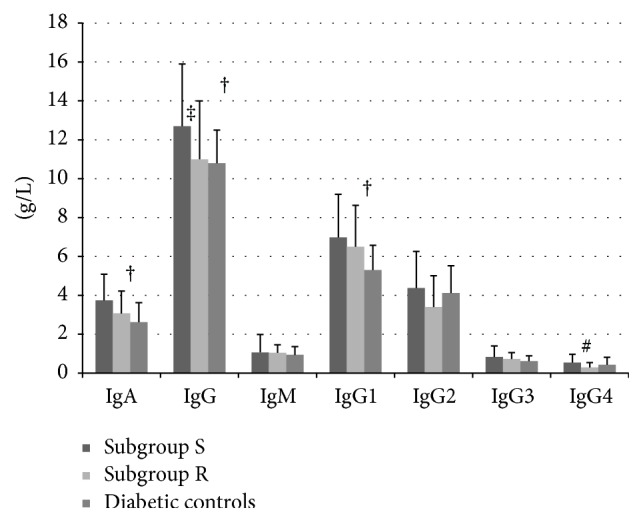
The comparison of serum levels of immunoglobulins including IgG subclasses among patients with chronic DFUs infected by sensitive and resistant pathogens and diabetic controls. Data are presented as means ± SD; patients with DFUs (diabetic foot ulcers) infected by sensitive (subgroup S; *n* = 50) or resistant pathogens (subgroup R; *n* = 18). Age, sex, and type of diabetes matched diabetic controls (*n* = 34); Ig: immunoglobulin; *p* value of significance between patients with DFUs infected by sensitive (subgroup S) or resistant pathogens (subgroup R) and diabetic controls determined using Kruskal-Wallis one way analysis of variance test. ^†^ *p* < 0.01: mean serum levels of IgA, IgG, and IgG1 in subgroup S versus diabetic controls. ^‡^ *p* < 0.05: mean serum levels of IgG in subgroup S versus subgroup R. ^#^ *p* < 0.01: mean serum levels of IgG4 in subgroup S versus subgroup R.

Patients infected by resistant pathogens differed significantly from those infected by sensitive microorganisms in the percentage of basophils (0.43 ± 0.24 versus 0.66 ± 0.38 × 10^9^/L; *p* < 0.01). Other measures of inflammation did not differ significantly between the study subgroups except a significantly higher percentage of NK cells in subgroup R (16.6 ± 7.5 versus 12.3 ± 5.7; *p* < 0.01) compared to subgroup S. Moreover, subgroup R did not reach high levels of IgA (3.07 ± 1.15 versus 3.74 ± 1.35 g/L; NS), IgG (11.01 ± 3.01 versus 12.69 ± 3.16 g/L; *p* < 0.05), and IgG1 (6.49 ± 2.13 versus 6.98 ± 2.22 g/L; NS) in contrast to subgroup S that differed significantly in mentioned parameters compared to diabetic controls (Figure 1). Subgroup R was characterised by significantly lower levels of IgG4 compared to subgroup S and diabetic controls (0.29 ± 0.25 versus 0.54 ± 0.43 and 0.43 ± 0.38 g/L; *p* < 0.01).

There were no significant differences in deficits of each immunological subclass between the study groups (Table 4), except a trend to higher IgG2 subclass deficiency shown in patients with infected DFUs compared to diabetic controls (22 versus 15%).

## 4. Discussion

In our study, we focused on the evaluation of systemic immunity changes to determine the occurrence of immune deficiencies at the level as of innate as of adaptive immunity. We aimed to clarify whether some patients with any kind of immunodeficiency exist among those treated for nonhealing chronically infected wounds. It could help in several cases potentially explain the prolonged DFUs healing and inadequate inflammatory response to local infection. We did not assess any immune parameters or mediators at the local level since their concentrations could be modified by a variety of factors including, for example, the mild forms of peripheral arterial disease or previous applications of biological active local dressings.

The main findings in patients with chronically infected DFUs were the significantly reduced percentages and absolute numbers of lymphocytes in contrast to diabetic controls without DFUs. We could explain the lower serum levels of lymphocytes by a previously described impairment of immune system at the level of lymphoid stem cells or mesenchymal cells [32] or other factors including stress [33], age [34], and possibly diabetes mellitus alone. Such factors could induce dysregulations of cytokine production [35] and glycation of specific proteins may be behind the control of production and differentiation of lymphocytes [32]. Moreover, decreased numbers of lymphocytes in patients with chronically infected DFUs could be related to an immunological pressure in which bacterial infection induces a production of polymorphonuclear cells potentially leading to relative suppression or increased apoptosis of lymphocytes as was described by Arya et al. [36]. Nevertheless, we did not find higher levels of neutrophils in our patients with DFUs.

The reduced number of lymphocytes seen in our study in patients with the DFUs was associated with a decline in the absolute numbers of almost all subpopulations of lymphocytes including T-helper, cytotoxic lymphocytes, B lymphocytes and naive, memory inactive, and effector cells as of CD4+ as ofCD8+ T lymphocytes. The reason for this finding is not still clear. Similar findings have been demonstrated in experimental animal models [37] and also in healthy individuals [38] under conditions of acutely induced hyperglycaemia, which led not only to lymphopenia but also to a decline of subpopulations of lymphocytes including CD3+, CD4+, and CD8+ T lymphocytes. In our study, however, correlations between blood glucose levels or HbA1c and subpopulations of lymphocytes were not seen.

Naive cells are defined by the expression of CD45RA antigen, which is present on the surface of approximately 50% of T lymphocytes. T lymphocytes expressing on their surface the CD45RO antigen are antigen activated cells (memory cells). CD45RO is usually present on the surface of approximately 40% of T cells (CD4+). During the activation of T lymphocytes (i.e., due to infection), the expression of CD45RA decreases simultaneously with the increase of the expression of CD45RO isoforms. The rise of effector cells, particularly CD8+ effector memory cells [3], together with the lower indexes of naive/memory cells should be seen during the infection. However, we observed neither increased counts of effector cells nor decreased indexes in patients with diabetes and chronic infection within the diabetic foot. We could speculate that the appropriate activation of selected types of lymphocytes does not occur due to the long-lasting depletion of the immune system by repeated and/or chronic infections.

Other significant changes that were found in our study were alterations in the humoral arm of the adaptive immune system, in immunoglobulins levels. In addition to elevated serum levels of total IgA and IgG, patients with DFUs had in comparison to diabetic controls significant differences in IgG subclasses, specifically increased levels of IgG1 and IgG3 suggesting activation of immune system by inflammation. Both molecules serve as complement activators via binding to the protein antigen of the pathogen. Therefore, the elevation of their values is not surprising in infection [3], although it has not been previously described in patients with chronically infected DFUs.

Since a response to bacterial infection could be altered in patients with DFUs, especially in the presence of immunoglobulin deficiencies particularly in IgG subclasses [1], we have also focused on their detection. A high number of patients from both study groups showed IgG2 deficiencies (defined as serum levels below 2.42 g/L), exceeding 15–20%. In the general population, although the most common selective deficit of IgG subclasses is an IgG2 deficit, however real prevalence data are unavailable. The occurrence of IgG subclass deficiencies in the diabetic population, even in patients with DFUs, has not been previously described. Since IgG2 plays an important role in the inflammatory cascade due to the ability to opsonise bacteria, its deficiency may contribute to an increased risk of infection [5, 6]. Detected deficits of IgG2 subclasses could possibly be secondary to diabetes, since serum levels of IgG2 and also IgG3 in our study negatively correlated with glycaemic control. We could thus hypothesise that reduced production or greater degradation of immunoglobulins as glycoproteins may be related to their glycation (B lymphocytes were reduced but their absolute numbers did not correlate with diabetes control). Conversely, deficits of certain immunoglobulin subclasses could be also caused by long-lasting consumption of them during chronic bacterial load. This theory is supported by the finding of decreased levels of IgG and predominantly of IgG4 subclasses in the patients repeatedly infected predominantly by resistant pathogens.

Looking at the possible alterations of the immune system in patients infected by resistant pathogens, we did not find the marked deficits of immune system that we initially supposed in such cohort in contrast to patients infected by sensitive microorganisms. Differences were seen predominantly in humoral immunity in the patients with resistant pathogens, in whom the serum levels of individual immunoglobulins and even IgG subclasses did not reach the levels detected in patients infected by sensitive microorganisms. In fact, IgG4 values were significantly lower, less than half of those found in the subgroup with sensitive pathogens and diabetic controls. We speculate that patients with DFUs infected by resistant pathogens have a predisposition to the long persistence of these bacteria possibly due to insufficiently activated humoral immunity, which in turn leads to reduced opsonisation, activation of cell-mediated immunity, and reduced elimination of pathogens by the immune system.

Our study did not show significant alterations of the immune system at the level of innate immune system including measures of phagocytosis. No changes were found in values of C3, which is usually reduced during infection due to its consumption [39] or hyperglycaemia [40]. Lower absolute numbers of NK cells in our study in patients with DFUs were probably given by lower values of total lymphocytes. This finding was also demonstrated in other studies [1, 41].

Significantly increased laboratory markers of infection in patients with infected DFUs are closely related to physiologically stimulated immune response to inflammation. This confirms the results of other studies [42, 43], which have not demonstrated dramatic increase of inflammation in patients with DFUs despite the presence of infectious complications including osteomyelitis. In our study, we found similarly to Weigelt et al. [44] only slightly elevated CRP levels up to 10 mg/L (detected in 33/68, 49%, of patients with the DFUs versus 5/34, 15%, of diabetic controls; *p* < 0.01), and more than half of the patients actually had CRP levels within the normal range. Elevated serum procalcitonin has been suggested to be a better predictor for the presence of infection [45] or osteomyelitis [43] than the other laboratory markers of inflammation, except CRP [46]. However, we did not find significant abnormalities in procalcitonin levels. From other laboratory markers of infection, the numbers of neutrophils were not significantly increased in our patients with DFUs, and this finding is in accordance with other studies [43, 46]. Based on the above data, we could surmise that a significant activation of systemic inflammatory immune response does not occur in patients with infected chronic DFUs as could be expected in the case of inflammation including osteomyelitis.

We conclude that in this group of patients with infected chronic DFUs mild activation of a systemic inflammatory response and significantly reduced numbers of lymphocytes, including nearly all of their subpopulations, were found. There was no evidence, however, of their activation, especially an increase of effector cells or reduction of appropriate subpopulations indexes (naive/memory cells) as may have been expected. In patients with infected DFUs we further demonstrated abnormalities of the humoral components of immunity particularly at the level of IgG subclasses combined with a relatively high incidence of their deficits, especially of IgG2. Changes in IgG subclasses were more prevalent in patients whose DFUs were infected by resistant pathogens.

We did not include into our study patients with noninfected diabetic foot ulcers since they represent only a minority group of our complicated patients with the diabetic foot in our foot clinic which provides a comprehensive care for patients from the whole republic, other centers, transplant and immunocompromised patients, and so forth [47]. In our previous study we focused on possible changes of innate or adaptive immunity and compared these results among patients with diabetic foot ulcers (without clinically relevant infection), diabetic controls, nondiabetics with leg ulcers, and healthy volunteers with no largely significant differences [21, 48, 49]. Therefore, we tried to select a group of patients with chronically infected nonhealed diabetic foot ulcers, where potential changes of the immune system will be more remarkable in contrast to match control group of diabetic patients. The results of our study showed that there are not major abnormalities of systemic immunity, and only certain deficits mainly at the level of lymphocytes subpopulations and immunoglobulin subclasses are present. Its effectiveness may be further reduced at the local level due to a number of factors, including the possible coexistence of mild capillary ischemia contributing to the development of DFUs [50] that could lead to worse migration of effector cells to the target destination.

We recommend performing more detailed immunological investigations in patients with DFUs with recurrent or chronic infectious complications particularly caused by resistant pathogens. These tests should focus on the determination of cell-mediated and humoral immunity, concretely on lymphocyte subpopulations and IgG subclasses.

## Figures and Tables

**Table 1:** A comparison of basic characteristics and certain inflammatory markers between the study groups.

Evaluated parameters	Patients with DFUs (*n* = 68)	Diabetic controls (*n* = 34)	*p* value
Age (years)	60.3 ± 7.7	58.5 ± 6.9	NS
Type of diabetes (type 1/type 2/other types; %)	16.2/80.9/2.9	15.6/84.4/0	NS
Serum glucose level (mmol/L)	10.8 ± 5.4	9.6 ± 3.7	NS
HbA1c according to IFCC (mmol/mol)	67 ± 19	62 ± 21	NS
Serum level of creatinine (*μ*mol/L)	107.7 ± 58.8	84.5 ± 14.3	*p* < 0.01
Serum level of CRP (mg/L)	10.7 ± 14.7	2.7 ± 2.2	*p* < 0.0001
Serum level of procalcitonin (*μ*mol/L)	0.07 ± 0.08	0.1 ± 0	NS

Data are presented as means ± SD; types of diabetes mellitus are in percentages; patients with DFUs (diabetic foot ulcers; *n* = 68); age, sex, and type of diabetes matched diabetic controls (*n* = 34); HbA1c: glycosylated hemoglobin; IFCC: International Federation of Clinical Chemistry; NS: nonsignificant; *p* value of significance between patients with DFUs and diabetic controls determined using *t*-test or Mann–Whitney test.

**Table 2:** The differences in selected parameters of innate immunity between the study groups.

Evaluated parameters	Patients with DFUs (*n* = 68)	Diabetic controls (*n* = 34)	*p* value
C3 (g/L)	1.32 ± 0.27	1.35 ± 0.19	NS
C4 (g/L)	0.3 ± 0.08	0.28 ± 0.07	NS
Percentage of phagocyting PMN cells (%)	98.2 ± 1.7	97.9 ± 1.4	NS
FAGSI	67.6 ± 27.7	65.8 ± 30.1	NS
Percentage of NK cells (%)	13.45 ± 6.46	13.15 ± 5.83	NS
Absolute numbers of NK cells (cells/*μ*L)	226 ± 113	327 ± 190	*p* < 0.01
CD14+HLA-DR (%)	80.9 ± 16.5	86.2 ± 14.7	NS

Data are presented as means ± SD; patients with DFUs (diabetic foot ulcers; *n* = 68); age, sex, and type of diabetes matched diabetic controls (*n* = 34); C: complement; PMN: polymorphonuclear; FAGSI: phagocyte stimulation index; NK: natural killers; CD: cluster of differentiation; NS: nonsignificant; *p* value of significance between patients with DFUs and diabetic controls determined using *t*-test or Mann–Whitney test.

**Table 3:** The differences in selected parameters of cell-mediated immunity between the study groups.

Evaluated parameters	Patients with DFUs (*n* = 68)	Diabetic controls (*n* = 34)	*p* value
% of total lymphocytes (calculated from blood counts)	23.08 ± 6.2	27.5 ± 6.9	*p* < 0.01
Absolute number of total lymphocytes (×10^9^/L)	1.78 ± 0.61	2.4 ± 0.83	*p* < 0.001
% of CD3+ lymphocytes	75.96 ± 7.64	75.77 ± 6.73	NS
Absolute number of CD3+ lymphocytes (cells/*μ*L)	1341 ± 484	1847 ± 669	*p* < 0.001
% of CD4+ lymphocytes	49.33 ± 8.74	47.24 ± 9.78	NS
Absolute number of CD4+ lymphocytes (cells/*μ*L)	870 ± 331	1122 ± 382	*p* < 0.01
% of CD8+ lymphocytes	25.4 ± 9.66	27.83 ± 10.84	NS
Absolute number of CD8+ lymphocytes (cells/*μ*L)	451 ± 252	712 ± 454	*p* < 0.01
% of CD19+ lymphocytes	10.1 ± 4.84	10.64 ± 4.11	NS
Absolute numbers of CD19+ lymphocytes (cells/*μ*L)	188 ± 124	251 ± 111	*p* < 0.01
% of CD4+ naive effector cells	1.43 ± 2.79	3.03 ± 5.45	NS
Absolute number of CD4+ naive effector cells (cells/*μ*L)	12.88 ± 27.42	34.72 ± 59.76	*p* < 0.05
% of CD4+ memory effector cells	21.74 ± 9.08	19.44 ± 8.64	NS
Absolute numbers of CD4+ memory effector cells (cells/*μ*L)	180 ± 85	220 ± 140	NS
Index of CD4+ naive/memory effector cells	0.09 ± 0.26	0.18 ± 0.38	NS
% of CD4+ naive inactive cells	22.8 ± 12.9	27 ± 13.4	NS
Absolute numbers of CD4+ naive inactive cells (cells/*μ*L)	207 ± 154	305 ± 194	*p* < 0.01
% of CD4+ memory inactive cells	54.3 ± 9.8	50.6 ± 10.5	NS
Absolute numbers of CD4+ memory inactive cells (cells/*μ*L)	472 ± 194	562 ± 214	*p* < 0.05
Index of CD4+ naive/memory inactive cells	0.47 ± 0.35	0.59 ± 0.37	NS
% of CD8+ naive effector cells	26.4 ± 17.5	24.1 ± 13.3	NS
Absolute number of CD8+ naive effector cells (cells/*μ*L)	128 ± 130	189 ± 180	*p* < 0.05
% of CD8+ memory effector cells	31.5 ± 11.2	32.4 ± 12.8	NS
Absolute number of CD8+ memory effector cells (cells/*μ*L)	145 ± 106	243 ± 260	*p* < 0.01
Index of CD8+ naive/memory effector cells	1.14 ± 1.28	1.02 ± 1.08	NS
% of CD8+ naive inactive cells	17.9 ± 10.1	21.9 ± 12.1	NS
Absolute number of CD8+ naive inactive cells (cells/*μ*L)	73.2 ± 47.4	144.2 ± 131.8	*p* < 0.001
% of CD8+ memory inactive cells	24.2 ± 10.4	21.7 ± 10.7	NS
Absolute number of CD8+ memory inactive cells (cells/*μ*L)	105 ± 72	136 ± 79	*p* < 0.05
Index of CD8+ naive/memory inactive cells	0.98 ± 1.02	1.31 ± 1.2	NS

Data are presented as means ± SD; patients with DFUs (diabetic foot ulcers; *n* = 68); age, sex, and type of diabetes matched diabetic controls (*n* = 34); CD: cluster of differentiation; NS: nonsignificant; *p* value of significance between patients with DFUs and diabetic controls determined using *t*-test or Mann–Whitney test.

**Table 4:** The differences in selected parameters of humoral immunity between the study groups.

Evaluated parameters	Patients with DFUs (*n* = 68)	Diabetic controls (*n* = 34)	*p* value
IgM (g/L)	1.1 ± 0.82	0.94 ± 0.42	NS
IgA (g/L)	3.56 ± 1.32	2.62 ± 1	*p* < 0.001
IgG (g/L)	12.24 ± 3.19	10.83 ± 1.69	*p* < 0.05
IgG1 (g/L)	6.85 ± 2.19	5.3 ± 1.28	*p* < 0.001
IgG2 (g/L)	4.12 ± 1.85	4.12 ± 1.4	NS
IgG3 (g/L)	0.81 ± 0.51	0.62 ± 0.27	*p* < 0.05
IgG4 (g/L)	0.47 ± 0.4	0.43 ± 0.38	NS
Deficit of IgG1 (<3,82 g/L)	2/68 (3%)	3/34 (9%)	NS
Deficit of IgG2 (<2,42 g/L)	15/68 (22%)	5/34 (15%)	NS
Deficit of IgG3 (<0,22 g/L)	0	0	NS
Deficit of IgG4 (<0,04 g/L)	3/68 (4%)	2/34 (6%)	NS

Data are presented as means ± SD; patients with DFUs (diabetic foot ulcers; *n* = 68); age, sex, and type of diabetes matched diabetic controls (*n* = 34); Ig: immunoglobulin; NS: nonsignificant; *p* value of significance between patients with DFUs and diabetic controls determined using *t*-test or Mann–Whitney test.
